# Involvement of Neutrophil Dynamics and Function in Exercise-Induced Muscle Damage and Delayed-Onset Muscle Soreness: Effect of Hydrogen Bath

**DOI:** 10.3390/antiox7100127

**Published:** 2018-09-25

**Authors:** Takuji Kawamura, Katsuhiko Suzuki, Masaki Takahashi, Miki Tomari, Reira Hara, Yuko Gando, Isao Muraoka

**Affiliations:** 1 Faculty of Sport Sciences, Waseda University, 2-579-15 Mikajima, Tokorozawa, Saitama 359-1192, Japan; tkawamura@aoni.waseda.jp (T.K.); tomari.miki@gmail.com (M.T.); imuraoka@waseda.jp (I.M.); 2 Waseda Bioscience Research Institute in Singapore, Waseda University, Singapore 138667, Singapore; m-takahashi@aoni.waseda.jp; 3 College of Sports Sciences, Nihon University, 3-34-1 Simouma, Setagaya, Tokyo 154-8513, Japan; hara.reira@nihon-u.ac.jp; 4 Department of Physical Activity Research, National Institutes of Biomedical Innovation, Health and Nutrition, 1-23-1 Toyama, Shinjuku, Tokyo 162-8636, Japan; gando-y@nibiohn.go.jp

**Keywords:** downhill running, muscle damage, delayed-onset muscle soreness, molecular hydrogen, neutrophil, oxidative stress, inflammation

## Abstract

The purpose of this study was to investigate the involvement of neutrophil dynamics and function in exercise-induced muscle damage (EIMD) and delayed-onset muscle soreness (DOMS), and the effect of molecular hydrogen (H_2_) intake on these parameters. Nine healthy and active young men performed H_2_ and placebo bath trial in a crossover design. They carried out downhill running (−8% slope) for 30 min at a speed corresponding to 75~85% of peak oxygen uptake (VO_2_peak). Subsequently, they repeated bathing for 20 min per day for one week. Degree of muscle soreness (visual analogue scale: VAS), peripheral leukocyte counts, neutrophil dynamics and function, muscle damage, and inflammation markers were measured. Plasma interleukin (IL)-6 concentration was significantly correlated with peripheral neutrophil count, VAS, and serum creatine kinase activity, respectively, after downhill running. Peripheral neutrophil count and serum myoglobin concentration were also significantly correlated. Conversely, there were no effects of H_2_ bath. These results suggest that IL-6 may be involved in the mobilization of neutrophils into the peripheral blood and subsequent EIMD and DOMS after downhill running; however, it is not likely that H_2_ bath is effective for the inflammatory process that is centered on neutrophils after downhill running.

## 1. Introduction

It is widely recognized in the sports science field that eccentric exercise and/or unaccustomed exercise causes muscle damage and delayed-onset muscle soreness (DOMS) [1,2]. These phenomena manifest at their peak at 24 to 72 h after cessation of exercise, and they eventually disappear by 5–10 days post-exercise [1,2]. Exercise-induced muscle damage (EIMD), which is one of the reasons for DOMS, is caused by not only mechanical factors, but also non-mechanical factors [3]. One possible mechanism causing muscle damage is the invasion of leukocytes, especially phagocytes, including neutrophils and macrophages, into skeletal muscle [4,5,6]. Phagocytes infiltrating muscle tissue play essential roles in tissue repair, such as protein degradation and removal of cellular debris, whereas activated phagocytes release pro-inflammatory cytokines and reactive oxygen species (ROS), and induce inflammation as well as myofiber membrane lysis [4,7]. In addition, ROS derived from phagocytes enhance gene expression of inflammatory cytokines in skeletal muscle, leading to further infiltration of phagocytes into damaged muscle tissue [4,7,8]. Thus, the production of inflammatory cytokines from the redox-sensitive pathway is also considered to be partly involved in muscle damage. Taken together, the inflammatory response and oxidative stress centered on phagocytes would be an important trigger in muscle damage and DOMS after eccentric exercise.

A number of previous studies have been investigated the influence of eccentric exercise, such as downhill running and resistance exercise on muscle damage [2]. In general, muscle damage is evaluated by leakage of muscle proteins (e.g., creatine kinase; CK and myoglobin; Mb) into the circulation [2]. Likewise, inflammation-related biomarkers (e.g., cytokines and leukocyte counts) and redox biomarkers (e.g., protein carbonyls and glutathione status) are frequently used as secondary physiological measurements [9,10]. In addition to these biomarkers, it is possible to evaluate various neutrophil functions, such as migration and ROS production as mediators and/or effectors by the luminol-dependent chemiluminescence (LmCL) [11,12,13,14,15,16,17]. Previous studies suggested that both migratory neutrophil count and its ROS productivity increased within a few hours after one-leg calf-raise exercise [11]. However, neutrophil functions evaluated by LmCL after systemic eccentric exercise, such as downhill running, have not been elucidated sufficiently. Since circulating neutrophil counts and systemic cytokine responses after dynamic eccentric exercise (i.e., downhill running) are different from static and local eccentric exercise (i.e., one-leg calf-raise) [11] and endurance exercise (i.e., cycling and level running) [12,13,14,15,16,17], the migratory neutrophil count and its ROS productivity may differ from both exercise modes. 

Several nutritional antioxidants such as vitamin C and vitamin E have been utilized as an intervention strategy to alleviate muscle damage and DOMS involving inflammation and oxidative stress. In fact, the ingestion of antioxidant substances has been suggested to alleviate muscle damage as well as exercise-induced inflammation and oxidative stress [18,19]. In addition to conventional antioxidants, it has been suggested that molecular hydrogen (H_2_) can act as an antioxidant. Although almost all studies investigated the effectiveness of H_2_ ingestion on various diseases and disease models that are related to oxidative stress [20,21], little is known about the influence of H_2_ on exercise-induced oxidative stress [22,23]. We previously reported that H_2_ ingestion after downhill running can be effective for the reduction of DOMS, whereas it did not affect the muscle damage, inflammation, and oxidative stress markers in blood [24]. However, we did not examine the influence on neutrophil dynamics and functions, which may explain the exercise-induced muscle damage, inflammatory processes, and DOMS.

Based on the above background, the main aim of the present study was to clarify the involvement of inflammatory markers, specifically neutrophil dynamics and function, in EIMD and DOMS after downhill running. Specifically, we quantified the migratory neutrophil count and its ROS productivity by using LmCL [11,12,13]. The secondary aim of this study was to investigate the effect of H_2_ baths after downhill running on neutrophil dynamics and function. H_2_ baths are the easiest and safest method of H_2_ intake along with drinking H_2_ water, but it is assumed that H_2_ bath can supply more H_2_ into the living body as compared to oral intake with limited drinking amount. In fact, H_2_ was reported to easily penetrate the skin and to be distributed throughout the body via blood flow within only 10 min, as judged by measuring H_2_ gas in expiration [20]. In addition, because H_2_ is orally taken into the body, the in vivo H_2_ concentration during bathing is always considered high.

## 2. Materials and Methods

### 2.1. Subjects

Ten healthy men aged between 20 and 30 years, who had a bathtub at home and had the habit of bathing every day, participated in this study. The subjects had a somewhat active lifestyle that enabled measurement of the maximum oxygen uptake (VO_2_peak) and completing the downhill running protocol. Those who were unable to restrict intense physical activity over the week of the experiment were excluded. Moreover, in accordance with the suggestion of previous reports that sex difference, especially sex hormones (i.e., estrogen), influences muscle damage, inflammation, and oxidative stress after eccentric exercise, the subjects of this study were limited to men [25]. Subjects received explanation of the purpose, contents, and risk of the experiment before participation in this study and then they signed informed consent and joined the experiment. Before the exercise test, a screening test consisting of height, body weight, percent body fat, resting 12-lead electrocardiogram, blood pressure and questionnaire about past medical history and symptoms was performed. In the screening test, those whose body mass index (BMI) values exceeded the range of 18 to 25 kg/m^2^, those who had abnormal resting 12-lead electrocardiography findings and blood pressure levels, and those who had relevant medical histories or symptoms were excluded from the present study. To the subjects who passed the screening test and confirmed that there were no health problems, the VO_2_peak was measured using a treadmill to determine exercise intensity in this experiment. As described in our previous study [24], the subjects’ characteristics were as follows (mean ± SD): age 25 ± 3 years, height 174.0 ± 3.3 cm, body weight 66.0 ± 6.7 kg, body fat 15.3 ± 4.2%, BMI 21.7 ± 2.0 kg/m^2^, seated systolic blood pressure 110 ± 8 mmHg, diastolic blood pressure 80 ± 6 mmHg, VO_2_peak 3566 ± 437 mL/min, and %VO_2_peak 54.4 ± 7.3 mL/kg/min. This research was conducted with the approval of the Ethics Committee of Waseda University (No. 2013-016), and was conducted according to the guidelines that were laid down in the Declaration of Helsinki. 

### 2.2. Measurement of Peak Oxygen Uptake

Subjects performed a progressive loading exercise using a treadmill until exhaustion in a laboratory set at room temperature 20 °C·50% humidity. The exercise load was started from a speed of 140 m/min with +1% slope, and the speed was gradually increased by 20 m/min every 2 min. The judgment of exhaustion was taken when the running speed could not be maintained despite encouragement from the experimenters. During exercise, rating of perceived exertion (RPE) by Borg scale, and heart rate were recorded every 2 min and electrocardiogram was monitored from the start to the end of exercise in order to ensure the safety of the subjects. Measurements of heart rate and electrocardiogram were performed by a bipolar dielectric method while using a monitor electrocardiograph (BSM-2401, Nihon Kohden, Tokyo, Japan). Oxygen uptake was measured by an energy metabolism analyzer (AE-310S, Minato Medical Science, Osaka, Japan), and the maximal value of oxygen uptake was set as VO_2_peak without judgment about whether it was VO_2_peak or not [24]. 

### 2.3. Experimental Protocol

This research was conducted as a placebo-controlled, randomized, single-blind, crossover study. On the first day of the experiment, balanced nutrition food (Calorie mate, Otsuka Pharmaceutical Co., Ltd., Tokushima, Japan) was taken with water 2 h before coming to the laboratory. After keeping rest for 1 h, subjective muscle pain was measured and then a blood sample was drawn from the antecubital vein by a physician or nurse. DOMS (thigh and leg muscles) was rated while using a visual analogue scale (VAS) that had a 100-mm line with “no pain” on one end and “extremely sore” on the other according to the previous study [11]. The subjects were asked to maintain a seated position and answer each questionnaire regarding the degree of pain (0–100 mm) felt at the state without muscle compression (VAS score of 1) and with strong muscle compression with their own hands (VAS score of 2). After the measurement of VAS and blood sampling, subjects carried out downhill running (−8% slope) for 30 min at a speed corresponding to 75~85% of VO_2_peak, which was preliminarily measured on flat running. H_2_ bath or placebo bath were performed for 20 min with a rest of 15 min after cessation of exercise, and VAS measurement and a blood sampling were performed again 5 min and 60 min after bath. To ensure the safety of the subjects, mineral water (200 mL) was given before and immediately after exercise and immediately after the end of bath. 

On the second day of the experiment, as in the first day, balanced nutrition food was taken with water for 2 h before coming to the laboratory, and VAS measurement and blood sampling were carried out after 30 min rest. On the third, fourth and eighth days of the experiments, measurements were carried out using the same protocol as the second day. Each subject continued bathing from the second day of the experiment to the seventh day (the day before the last measurement). All of the participants carried out the other trial with at least a one week interval (mean ± SD: 22 ± 14 days). 

### 2.4. Bath Methods

2.5 g of magnesium hydride (MgH_2_) or 5.5 g magnesium hydroxide agent (Mg(OH)_2_) with equivalent amount of Mg was dissolved in hot water capable of bathing the whole body (200 L). Subjects repeated baths for 10 min twice with a 3 min rest between them. Baths were done in the laboratory on the first day of the experiment and at the subject’s home from the second to seventh days of the experiment. We handed over MgH_2_ or Mg(OH)_2_ to the subjects and instructed verbally, as follows: (1) dissolve bath agent 10 min before bathing, (2) perform whole body bathing, and (3) measure hot water temperature. For the bath on the first day of the experiment, bathing was carried out 15 min after the cessation of exercise, while taking into consideration that troubles might be caused by body temperature elevation and skin vasodilatation. From the result of H_2_ concentration in exhaled gas, it is believed that H_2_ is distributed throughout the body 10 min after H_2_ bathing [21].

### 2.5. Blood Sampling and Analyses

Approximately 12 mL of blood drawn into a syringe was quickly transferred to blood sampling tubes (Termo, Tokyo, Japan). Immediately after blood sampling, complete blood cell counts were determined in a portion of ethylene diamine tetraacetic acid (EDTA)-treated whole blood by using an automatic blood cell counter (PocH100i, Sysmex, Kobe, Japan). To obtain plasma or serum, the blood samples collected into blood sampling tubes with or without EDTA, respectively (Termo, Tokyo, Japan), were centrifuged at 3000 rpm at 4 °C for 10 min and then stored at −80 °C until analysis. Interleukin (IL)-6 was measured using IL-6 high-sensitivity ELISA kit (R&D, Minneapolis, MI, USA). On the other hand, analyses of CK activity and Mb concentration were conducted by external institution (BML, Kawagoe, Saitama, Japan). Measurements using blood samples that were entrusted to the external institution for each experiment were performed within several weeks. On the other hand, IL-6 level was measured within several months after the completion of all the experiments.

### 2.6. Neutrophil Functions

Neutrophil functions were measured as described previously [13,26]. Specifically, peripheral blood samples were drawn from subjects using 2 mL Na-heparin tubes (Terumo, Tokyo, Japan). The blood samples were mixed with 2.5 mM luminol (5-amino-2,3-dihydro-1,4-phthalazinedione; Sigma Aldrich, St. Louis, MO, USA) at a ratio of 1:1. Then, 150 μL luminol-blood samples were layered on 50 μL S-TGP gel that was prepared in a tube at 37 °C and it was promptly measured by LmCL (relative light unit: RLU) using a luminometer (Gene Light 55, Microtec, Funabashi, Japan). The samples were incubated at 37 °C and the production of ROS from neutrophils was monitored in a kinetic mode for 60 min. After measurement of LmCL at 60 min, luminol-blood samples were removed and the tubes with 50 μL S-TGP gel in which neutrophils migrated were washed three times with PBS warmed at 37 °C. Then, the tubes with gel were cooled on ice, and 50 μL Turk solution (Wako, Osaka, Japan) were added and mixed well. The liquids obtained in this way were set on the C-Chip (Disposable haemocytometer, Neubauer improved, DHC-No.1, Digital Bio, Seoul, Korea), and the migratory cell number was counted under the microscope. Migrated neutrophil number was calculated by 20 times multiplication of the counted cell number.

### 2.7. Statistical Analysis

In this study, one subject was found to have a history of immunoglobulin A nephropathy after the experiment was completed. The baseline muscle soreness (VAS) and inflammatory marker (IL-6) value were extremely higher than those in the other nine subjects. Thus, the data of nine subjects, excluding that one subject, were used for data analysis. Data were presented as the means ± standard deviations (SD). The differences between the groups in the exercise intensity (running speed, VO_2_, %VO_2_peak, and heart rate) and bath temperature of one week were determined using an un-paired *t*-test. Data were tested for sphericity using the Mauchly test. If the homogeneity assumption was violated, we used the Greenhouse-Geisser and adjustment. Peripheral leukocyte counts, neutrophil dynamics and function were analyzed using two-way analysis of variance (ANOVA) for repeated measures. When a significant main effect of time was observed, we used the Bonferroni method for post hoc comparisons. At the measurement points where significant changes from the baseline values were observed, relationships were analyzed using Pearson’s correlation coefficient. Specifically, the rates of the changes from the baseline values of the peripheral neutrophils–2 h, neutrophils/lymphocytes–2 h, CK–Post, CK–2 h, Mb–Post, Mb–2 h, IL-6–Post, and IL-6–2 h, and the amounts of changes from the baseline values of VAS 1–4 h, VAS 1–48 h, VAS 2–24 h, and VAS 2–48 h were used for the analysis. Statistical analysis was performed with SPSS (ver. 24 for Windows; SPSS Inc., Chicago, IL, USA), and *p* < 0.05 was considered as statistically significant.

## 3. Results

### 3.1. Exercise Intensity during Downhill Running and Bath Temperature

As in our previous study [24], exercise intensity during downhill running was not significantly different between the trials, such as running speed (H_2_ trial: 206 ± 34 m/min; Placebo trial: 205 ± 34 m/min), VO_2_ (H_2_ trial: 2003 ± 320 mL/min; Placebo trial: 2028 ± 316 mL/min), %VO_2_peak (H_2_ trial: 56.0 ± 3.2%; Placebo trial: 56.7 ± 3.4%), and heart rate (H_2_ trial: 154 ± 10 bpm; Placebo trial: 154 ± 11 bpm). Similarly, there was no significant difference between the trials at the average bath temperature of one week (H_2_ trial: 39.1 ± 1.2 °C; Placebo trial: 38.9 ± 1.0 °C). 

### 3.2. Peripheral Leukocyte Counts

**Figure 1:**
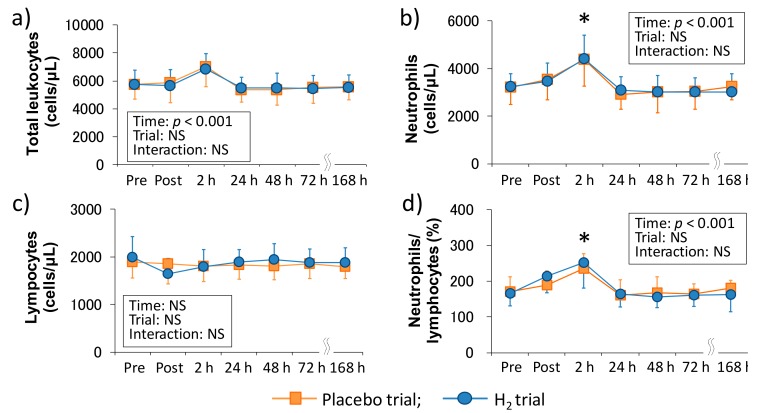
Time-course changes of peripheral leukocyte counts after downhill running. (**a**) Total leukocyte count, (**b**) Neutrophil count, (**c**) Lymphocyte count, and (**d**) Neutrophils/lymphocytes ratio. Pre, Pre exercise; Post, approximately 40 min after exercise (end of bath), 2, 24, 48, 72, and 168 h after exercise. Data are presented as the means ± standard deviations (SD) of nine subjects in each trial (placebo: *n* = 9, H_2_ trial: *n* = 9). There was a significant time effect in the total leukocyte count (*p* < 0.001), neutrophil count (*p* < 0.001), and neutrophils/lymphocytes ratio (*p* < 0.001) (two-way ANOVA for repeated measures). * indicates a significant change from the baseline value.

Figure 1 shows the time-course change of peripheral leukocyte counts following downhill running in each trial. There was a main effect of time in the total leukocyte count (*p* < 0.001), neutrophil count (*p* < 0.001), and neutrophils/lymphocytes ratio (*p* < 0.001), and neutrophil count and neutrophils/lymphocytes ratio were significantly increased 2 h after exercise (*p* < 0.05, respectively); however, there was no main effect of time in the peripheral lymphocyte count. Also, the main effect of trial and interaction were not significant in all of these markers. 

### 3.3. Neutrophil Dynamics and Function

**Figure 2:**
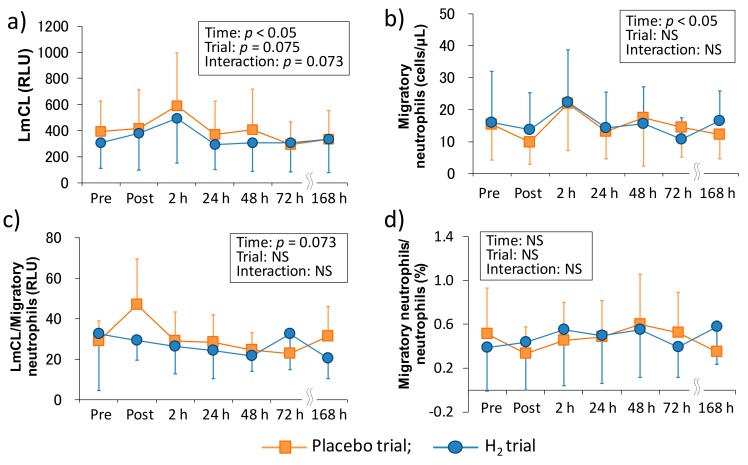
Time-course changes of neutrophil functions after downhill running. (**a**) Reactive oxygen species productivity of neutrophils, (**b**) migratory neutrophils count, (**c**) reactive oxygen species productivity of neutrophils/migratory neutrophils ratio, and (**d**) migratory neutrophils count/peripheral neutrophil count ratio. LmCL, luminal-dependent chemiluminescence; RLU, relative light unit; Pre, Pre exercise; Post, approximately 40 min after exercise (end of bath), 2, 24, 48, 72, and 168 h after exercise. Data are presented as the means ± standard deviations (SD) of nine subjects in each trial (placebo: *n* = 9, H_2_ trial: *n* = 9). There was a significant time effect in the reactive oxygen species productivity of neutrophils (*p* < 0.05), and the migratory neutrophils count (*p* < 0.05) (two-way ANOVA for repeated measures).

Figure 2 shows the time-course change of neutrophil dynamics and functions in each trial. There was a main effect of time in the LmCL (*p* < 0.05), which is represented as ROS productivity of neutrophils, and the main effects of trial and interaction tended to be significant (*p* = 0.075 and *p* = 0.073, respectively). In the migratory neutrophil count, there was a main effect of time (*p* < 0.05). On the other hand, although LmCL/migratory neutrophils ratio showed an increasing trend in the main effect of time (*p* = 0.073), there was no main effect of time in the migratory neutrophils/neutrophils ratio. Also, the main effect of trial and interaction were not significant in the migratory neutrophil count, LmCL/migratory neutrophils ratio, and migratory neutrophils/neutrophils ratio.

### 3.4. Relationships among Muscle Soreness, Muscle Damage and Inflammatory Markers

In accordance with a previous study [11], the relationships among the VAS score for muscle soreness, muscle damage and inflammatory markers were investigated on the basis of changes from the baseline. As shown in Table 1, both serum CK activity and Mb concentration as muscle damage markers at each time point were significantly correlated with VAS 1 or VAS 2. More specifically, CK–Post and VAS 2–48 h (*r* = 0.46, *p <* 0.05), Mb–Post and VAS 1–48 h (*r* = 0.55, *p* < 0.05), Mb–Post and VAS 2–24 h *(r* = 0.58, *p* < 0.05), Mb–2 h and VAS 1–24 h (*r* = 0.54, *p* < 0.05), Mb–2 h and VAS 1–48 h (*r* = 0.59, *p* < 0.01), and Mb–2 h and VAS 2–24 h (*r* = 0.62, *p* < 0.01) were significantly correlated, respectively. There were significant correlations between the plasma IL-6–Post and VAS 1–48 h, and VAS 2–48 h (*r* = 0.50, *p* < 0.05 and *r* = 0.62, *p* < 0.01, respectively). Regarding the associations between muscle damage and inflammatory markers, there were significant correlations between the neutrophil count–2 h and Mb–Post (*r* = 0.57, *p* < 0.05), neutrophil count–2 h and IL-6–Post (*r* = 0.69, *p* < 0.01), CK–Post, and IL-6–Post (*r* = 0.77, *p* < 0.001).

## 4. Discussion

**Figure 3:**
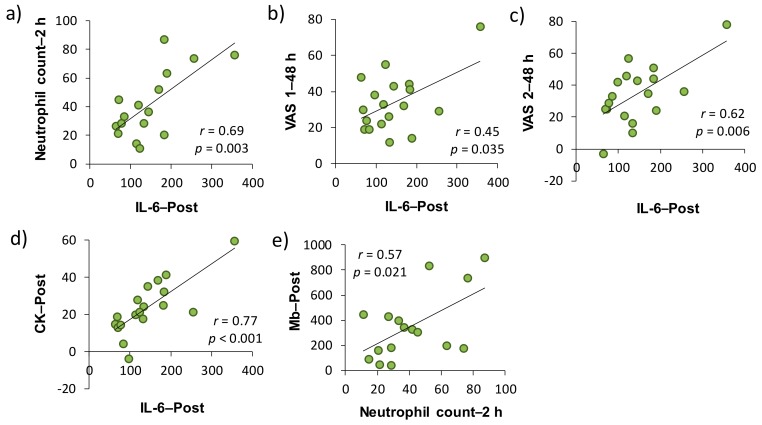
Relationships among the changes in muscle soreness, muscle damage, and inflammatory markers from the baseline in placebo and H_2_ trials (Pearson’s correlation coefficient). Subfigures show the relationships between (**a**) IL-6–Post and Neutrophil count–2 h, (**b**) IL-6–Post and VAS 1–48 h, (**c**) IL-6–Post and VAS 2–48 h, (**d**) IL-6–Post and CK–Post, (**e**) Neutrophil count–2 h and Mb–Post. IL, interleukin; VAS, visual analogue scale; CK, creatine kinase; Mb, myoglobin. Post, approximately 40 min after exercise (end of bath), 2 and 48 h after exercise.

We investigated the involvement of inflammatory markers, especially neutrophil dynamics and function, in EIMD and DOMS after downhill running. We also examined the effects of H_2_ baths after downhill running on neutrophil dynamics and function. As a result, IL-6–Post was significantly correlated with neutrophil count–2 h, VAS 1–48 h, VAS 2–48 h, and CK–Post, respectively, and there was a significant correlation between neutrophil count–2 h and Mb–Post (Figure 3). These findings suggested that IL-6 was involved in the mobilization of neutrophils into peripheral blood, which may be associated, at least partly, with EIMD and DOMS after downhill running. Conversely, the present study showed that H_2_ baths after downhill running did not influence the neutrophil dynamics and function.

Previous studies suggested that the infiltration of neutrophils into tissues causes subsequent inflammation and oxidative stress and is also partially involved in muscle damage and DOMS [6,7,13,18,27]. Therefore, measuring the dynamics and function of neutrophils are crucial in elucidating the mechanism of the subsequent chain reactions. In this study, we evaluated neutrophil functions, such as migration and ROS productivity ex vivo by using LmCL. Kanda et al. (2013) reported that migratory neutrophils count significantly increased 4 h after one-leg calf-raise exercise and its ROS productivity tended to be significant (*p* = 0.07) at the same time point [11]. In the present study, there was a main effect of time in the migration and ROS productivity of neutrophils, and these markers peaked at 2 h after downhill running (Figure 2). Despite the non-eccentric exercise model, another study showed that these markers significantly increased immediately after (migratory neutrophil count) or three hours after (ROS productivity by neutrophils) duathlon race [12]. These findings suggested that peripheral neutrophils may infiltrate into the damaged tissue within several hours, regardless of the types of muscle-damaging exercise, and cause inflammatory reactions by producing ROS [13]. 

In comparison with the same exercise mode (i.e., downhill running), Peake et al. (2005) reported that downhill running at a moderate intensity (60%VO_2_max) stimulates greater increases in circulating muscle proteins and enzymes (i.e., CK and Mb), plasma cytokines (i.e., IL-6), and peripheral total leukocyte and neutrophil counts, whereas no significant main effect of time was observed in the ROS productivity of neutrophils that were measured by in vitro stimulation after downhill running [10]. In the present study, although we showed almost similar results as the previous study, increase in migration and ROS productivity of neutrophils showed significant main effects (Figure 2). The difference in these results was probably due to the measurement methodology. More specifically, a previous study measured ROS productivity of neutrophils separated by centrifugation from whole blood, but we measured it without the separation procedure by overlaying fresh whole blood on the hydrogel [11,12,13,26]. In this method, unlike the conventional methods, it is considered that the migration and ROS productivity of neutrophils can be measured without changing neutrophil functions by the separation process after blood sampling [28]. However, it may be premature to mention the differences in measurement methodology used in the present research, and the effects of the differences between the two measurement methodologies must be directly compared.

The present study demonstrated that plasma IL-6 was significantly correlated with muscle soreness, as assessed by VAS. In addition, peripheral neutrophil count and plasma IL-6 concentration were significantly correlated with indirect muscle damage markers (i.e., CK and Mb) (Table 1, Figure 3). A number of studies have attempted to clarify the involvement of inflammatory markers in EIMD and DOMS by comparing the time-course changes in each indicator after eccentric exercise [11,29,30,31,32]. However, the evidence for the role of inflammation in EIMD and DOMS is limited and their causal relationship has not been elucidated yet. In this study, changes from the baseline of some inflammatory markers (i.e., peripheral neutrophil count and IL-6 level) have been shown to be a predictor of subsequent muscle damage and pain, but the issue whether inflammation is a cause or consequence of EIMD and DOMS after eccentric exercise has not been clarified. On the other hand, changes from the baseline of efflux proteins, such as serum CK activity and Mb concentration, significantly correlated with changes in VAS 1 and 2 (24 and 48 h after downhill running). Taken together, our findings suggested that inflammation contributed to, at least partly, both EIMD and DOMS, and efflux proteins, especially in Mb, were strongly associated with DOMS after downhill running. 

H_2_ is a colorless, odorless, and tasteless gas with high flammability and low abundance in the atmosphere [33]. Recently, it has been reported that H_2_ can improve various diseases such as metabolic diseases, cardiovascular diseases and neurodegenerative diseases via several physiological functions including anti-oxidative and anti-inflammatory effects [21,22]. In the sports science field, although several studies have been reported on the influence of H_2_ intake on exercise-induced oxidative stress and other biomarkers (e.g., inflammatory markers, muscle damage markers, and energy substrates), the findings on the effectiveness of H_2_ were fragmentary [23,24,25,34]. Therefore, we investigated the effects of H_2_ baths after downhill running on neutrophil dynamics and functions. Consequently, our results showed that H_2_ baths did not affect the peripheral leukocytes and neutrophil dynamics and functions (Figure 1 and Figure 2). We previously reported that H_2_ baths after downhill running did not affect the cytokine response (i.e., IL-6 and IL-17a) and oxidative stress markers (e.g., malondialdehyde, derivatives of reactive oxygen metabolites, biological antioxidant potential) [24]. These results suggested that H_2_ baths after downhill running is not effective on the inflammatory process that is centered on neutrophils after downhill running. However, because the most effective method of H_2_ intake is still unknown and the effect of H_2_ on exercise-induced inflammation using a different intake method may need to be examined.

This study has several limitations. First, we performed a correlation analysis for each variable using change from the baseline. However, these results of analyses used the data obtained by the same subject performing two downhill exercises, and thus the influence by “repeated bout effect” would have been inevitable [15,16,35]. Therefore, the plot point may have approached the origin of the figure (Figure 3). In addition, the effect of H_2_ bath was not statistically significant, but it may have influenced each variable a little. Secondly, our exercise setting based on preliminary VO_2_peak test was considerably lower than expected, as shown in our previous report [24]. Therefore, it may have been inadequate as a stimulus to young healthy men, which is considered to have caused no difference between the local (e.g., one-leg calf-raise) and systemic (e.g., downhill running) inflammatory reactions. In addition to the exercise intensity, exercise duration may have influenced the slight change in the overall number of cells (e.g., total leukocytes and lymphocytes) after downhill running. In fact, the training status of the subjects were different, but the increases in the peripheral total leukocyte and neutrophil counts observed in this study were lower than those after 45 min of downhill running at approximately the same exercise intensity in the previous study [10]. Taken together, to clarify the differences in inflammatory response, especially neutrophil dynamics and functions, after dynamic eccentric exercise, static and local eccentric exercise, and endurance exercise, it would be important to set the exercise intensity and duration more rigorously.

## 5. Conclusions

The present study revealed that plasma IL-6 was associated with peripheral neutrophil count, VAS 1 and 2, serum CK activity, respectively, after downhill running. In addition, it was shown that peripheral neutrophil count and serum Mb concentration was related in this study. Conversely, H_2_ baths after downhill running had no effects on peripheral neutrophil count, and both dynamics and functions of neutrophils. These findings suggest that IL-6 might be involved, at least partly, in the mobilization of neutrophils into the peripheral blood and subsequent muscle damage and muscle pain after downhill running [11,12,13,26]; however, the intake of H_2_ as anti-oxidative and anti-inflammatory agent is likely not to be effective on the inflammatory process via neutrophils after downhill running.

## Figures and Tables

**Table 1:** Relationships among muscle soreness, muscle damage and inflammatory markers after downhill running in placebo and H_2_ trials.

		Neutrophils	Neutrophils/Lymphocytes	VAS 1		VAS 2		CK		Mb		IL-6	
		2 h	2 h	24 h	48 h	24 h	48 h	POST	2 h	Post	2 h	Post	2 h
Neutrophils	2 h		0.376	−0.159	0.173	0.070	0.326	0.480	−0.030	0.571 *	0.413	0.685 **	0.435
Neutrophils/lymphocytes	2 h	0.376		−0.003	0.307	−0.145	−0.093	0.248	−0.174	0.423	0.359	0.256	0.165
VAS 1	24 h	−0.159	−0.003		0.833 ***	0.720 **	0.588 *	0.178	0.225	0.449	0.541 *	0.212	0.215
	48 h	0.173	0.307	0.833 ***		0.570 *	0.654 **	0.426	0.240	0.553 *	0.593 **	0.498 *	0.005
VAS 2	24 h	0.070	−0.145	0.720 **	0.570 *		0.816 ***	0.271	0.249	0.580 *	0.624 **	0.289	0.023
	48 h	0.326	−0.093	0.588 *	0.654 **	0.816 ***		0.475 *	0.237	0.415	0.413	0.624 **	0.257
CK	Post	0.480	0.248	0.178	0.426	0.271	0.475 *		0.637 **	0.439	0.440	0.768 ***	0.334
	2 h	−0.030	−0.174	0.225	0.240	0.249	0.237	0.637 **		0.346	0.436	0.323	0.179
Mb	Post	0.571 *	0.423	0.449	0.553 *	0.580 *	0.415	0.439	0.346		0.971 ***	0.423	0.019
	2 h	0.413	0.359	0.541 *	0.593 **	0.624 **	0.413	0.440	0.436	0.971 ***		0.369	0.058
IL-6	Post	0.685 **	0.256	0.212	0.498 *	0.289	0.624 **	0.768 ***	0.323	0.423	0.369		0.547 *
	2 h	0.435	−0.165	−0.215	−0.005	−0.023	0.257	0.334	0.179	0.019	0.058	0.547 *	

VAS, visual analogue scale; CK, creatine kinase; Mb, myoglobin; IL, interleukin. Post, approximately 40 min after exercise (end of bath), 2, 24, and 48 h after exercise. *, **, and *** represent significant correlations at levels of *p* < 0.05, *p* < 0.01 and *p* < 0.001, respectively.
